# Interferon‐stimulated gene products as regulators of central carbon metabolism

**DOI:** 10.1111/febs.15625

**Published:** 2020-12-01

**Authors:** Kourosh H. Ebrahimi, Javier Gilbert‐Jaramillo, William S. James, James S.O. McCullagh

**Affiliations:** ^1^ Chemistry Research Laboratory Department of Chemistry University of Oxford UK; ^2^ Sir William Dunn School of Pathology University of Oxford UK; ^3^ Department of Physiology, Anatomy and Genetics University of Oxford UK

**Keywords:** GAPDH, immunometabolism, ISG, viperin, viruses

## Abstract

In response to viral infections, the innate immune system rapidly activates expression of several interferon‐stimulated genes (ISGs), whose protein and metabolic products are believed to directly interfere with the viral life cycle. Here, we argue that biochemical reactions performed by two specific protein products of ISGs modulate central carbon metabolism to support a broad‐spectrum antiviral response. We demonstrate that the metabolites generated by metalloenzymes nitric oxide synthase and the radical S‐adenosylmethionine (SAM) enzyme RSAD2 inhibit the activity of the housekeeping and glycolytic enzyme glyceraldehyde 3‐phosphate dehydrogenase (GAPDH). We discuss that this inhibition is likely to stimulate a range of metabolic and signalling processes to support a broad‐spectrum immune response. Based on these analyses, we propose that inhibiting GAPDH in individuals with deteriorated cellular innate immune response like elderly might help in treating viral diseases such as COVID‐19.

## Graphical abstract

ISG products are believed to directly block viral replication. We propose an alternative mechanism based on emerging evidence. We discuss that the metabolic products of two ISG proteins, namely RSAD2 and NOS, inhibit activity of GAPDH to regulate central carbon metabolism and support a broad‐spectrum antiviral immune response via at least four mechanisms: eicosanoids storm, antigen cross‐presentation via MHC‐I, modulating NFAT and NF‐κB pathways, and S‐nitrosylation of viral proteins.

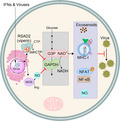

**Abbreviations**
1,3‐BPG: 1,3‐biphosphoglycerate
COVID‐19: coronavirus disease 2019
cADPR: cyclic ADP‐ribose
ddhCTP: 3'‐deoxy‐3',4'‐didehydro analogue
GAPDH: glyceraldehyde 3‐phosphate dehydrogenase
G3P: glyceraldehyde 3‐phosphate
hiPSCs: human induced pluripotent stem cells
ISG: interferon‐stimulated gene
NFAT: nuclear factor of activated T cells
NOS: nitric oxide synthase
RSAD2: radical S‐adenosylmethionine domain‐containing protein 2
RdRps: RNA‐dependent RNA polymerases
SAM: S‐adenosylmethionine

## Introduction

**Fig. 1:**
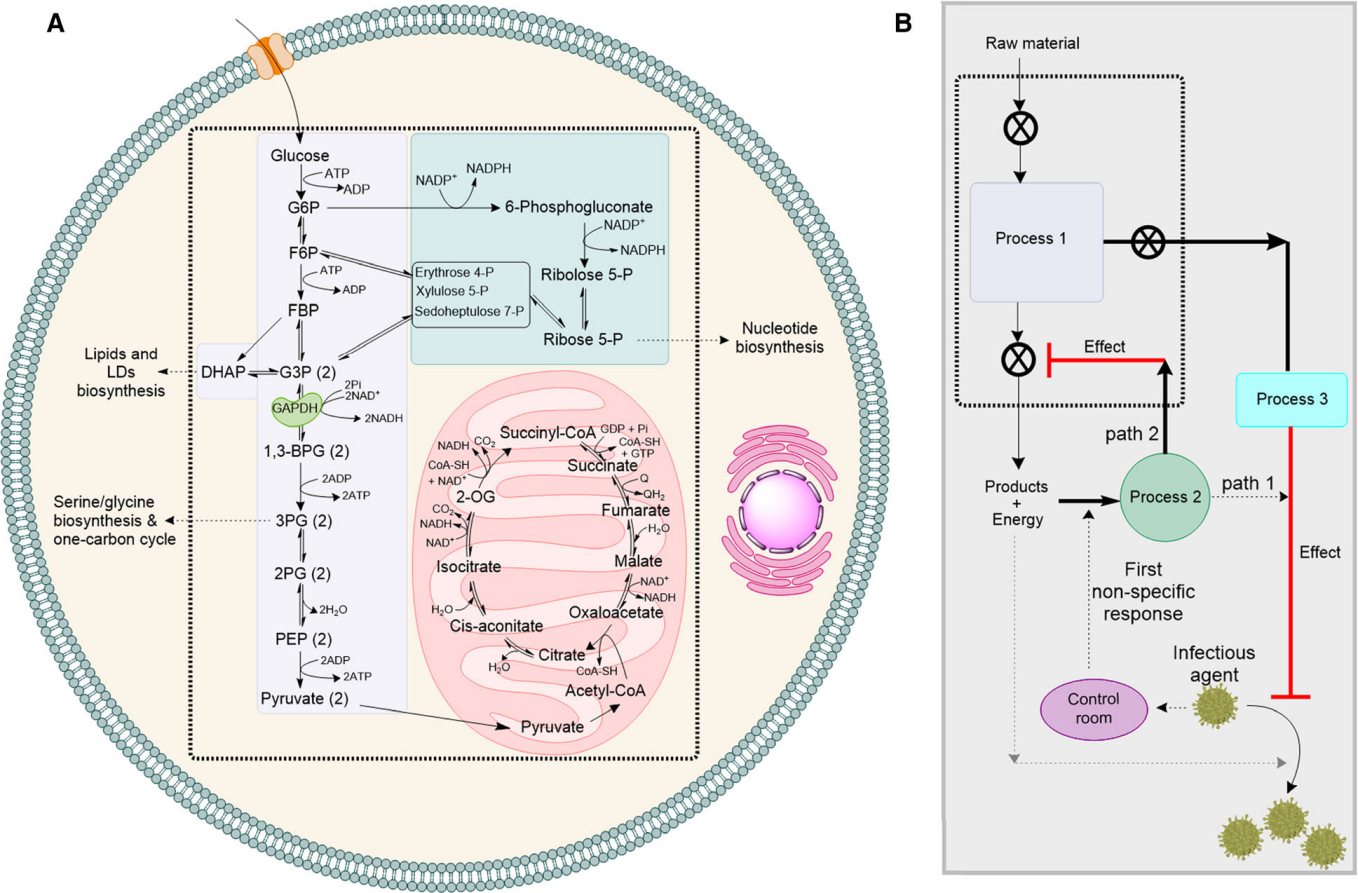
Central carbon metabolism and viral infection. (a) Central carbon metabolism (glycolysis, pentose phosphate pathway and TCA cycle) converts sugars to the building blocks of DNA and RNA, proteins and lipids. Additionally, it generates energy in the form of ATP and redox cofactors NAD^+^/NADH and NADP^+^/NADPH. Abbreviations: LDs, lipid droplets; G6P, glucose 6‐phosphate; F6P, fructose 6‐phosphate; FBP, fructose 1,6‐biphosphate; G3P, glyceraldehyde 3‐phosphate; DHAP, dihydroxyacetone phosphate; 1,3‐BPG, 1,3‐biphosphoglycerate; 3PG, 3‐phosphoglycerate; 2PG, 2‐phosphoglycerate; PEP, phosphoenolpyruvate; 2‐OG, 2‐oxoglutarate. (b) A systems engineering analogy describing function of the protein products of ISGs as (1) direct effectors of viral replication and (2) in the remodelling of central carbon metabolism to support broad‐spectrum immune response. The cell is like a factory and central carbon metabolism is the process 1. If an infectious agent enters the factory, the first response of the control room would be to use some of the available products and energy (process 2) to either directly inhibit viral replication (path 1) or to inhibit the production process (path 2). The outcomes of path 2 will be (i) reduction in formation of products and energy to limit access of pathogen to these resources and (ii) support of process 3, which restricts replication of the infectious agent.

Central carbon metabolism converts sugars into a range of metabolic precursors that are used to generate biomass and energy required for the cellular function [1] (Fig. 1A). Consequently, remodelling of central carbon metabolism occurs in many human diseases such as cancer [2] and is at the forefront of the host–pathogen interactions. Pathogens like bacteria or viruses are dependent on host cellular metabolites and proteins to support their reproduction. To fight viral infections, all cells are equipped with a nonspecific response consisting of the expression of several proteins and enzymes, induced by different types of interferons, and thus, are referred to as interferon‐stimulated gene (ISG) products. Most previous studies have led to the conclusion that the protein products of these genes directly act on the viral life cycles to restrict their replication [3, 4]. On the contrary, we propose a new model based on available data in the literature and an analogy from a system engineering perspective (Fig. 1B): A cell can be considered as a factory and central carbon metabolism as the main process for converting a raw material to products and energy for the factory to function (Fig. 1B). When an infectious agent enters the factory, it will highjack the main process and use the products for its reproduction. Under this circumstance, the first response of the control room would be to use some of the available products in a second reaction (analogous to the function of ISGs) to directly block viral replication and to inhibit the main process. This would limit the nutrients for the reproduction of the infectious agent, while redirecting the materials and energy to a third process that can eliminate the invading agent. Accordingly, we suggest that the metabolites generated by some ISG proteins contribute to the remodelling of the central carbon metabolism in support of a broad‐spectrum antiviral immune response. We discuss emerging evidence that supports this model. We show how the early metabolites generated by the biochemical reactions of two ISG metalloenzymes, namely nitric oxide synthase (NOS) and the radical‐SAM enzyme RSAD2, inhibit the glycolytic enzyme glyceraldehyde 3‐phosphate dehydrogenase (GAPDH) and how this inhibition is likely to support a broad‐spectrum antiviral immune response.

## Mechanisms adopted by cells to inhibit GAPDH

GAPDH is a housekeeping protein catalysing a critical step in glycolysis, with additional functions in DNA repair [5], cytoskeletal dynamics and vesicular trafficking between cellular compartments [6] and redox signalling and apoptosis [7]. As a glycolytic enzyme, it catalyses the NAD^+^‐dependent transformation of glyceraldehyde 3‐phosphate (G3P or GAP) to 1,3‐biphosphoglycerate (1,3‐BPG) (Fig. 1A). Metabolomic analysis together with computational studies have revealed that flux through GAPDH is a rate‐limiting step in glycolysis [8]. Here, we discuss mechanisms adopted by the cellular innate immune response to inhibit NAD^+^‐dependent conversion of G3P by GAPDH, focussing on those associated with ISG products specifically rather than other mechanisms such as malonylation [9].

### Inhibition of GAPDH by ddhCTP ribonucleotide analogue generated by RSAD2 (viperin)

**Fig. 2:**
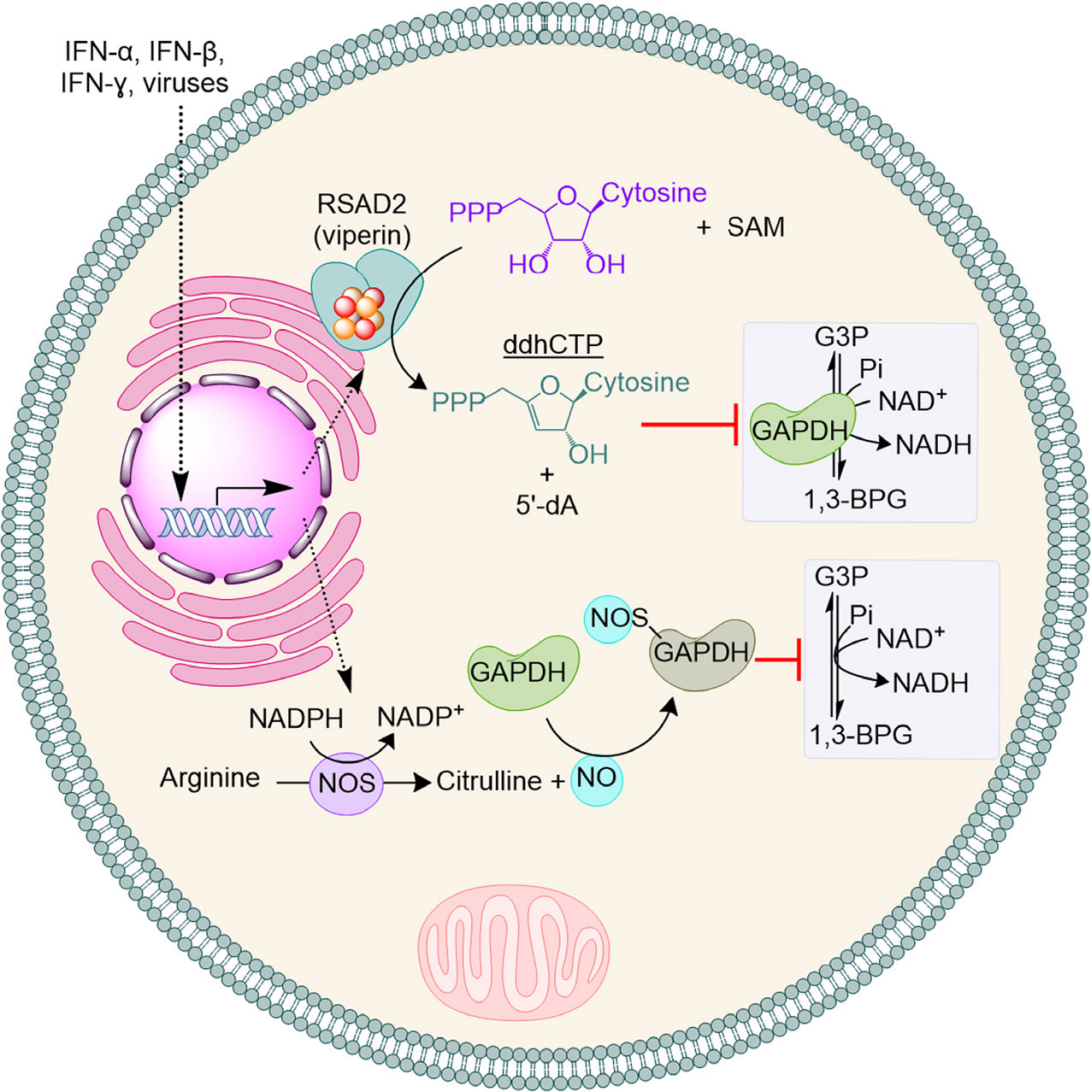
The early response of the cellular innate immune system inhibits NAD^+^‐dependent activity of GAPDH. In response to interferons, viruses or bacteria, the cells express metalloenzymes RSAD2 (viperin) and/or nitric oxide synthase (NOS). RSAD2 uses S‐adenosylmethionine (SAM) to catalyse transformation of CTP to ddhCTP, which inhibit activity of GAPDH. 5´‐deoxyadenosine (5´‐dA) is formed as a by‐product. On the other hand, NOS generates NO, which induces S‐nitrosylation of GAPDH and inhibits its activity.

Radical S‐adenosylmethionine (SAM) domain‐containing protein 2 (RSAD2) also known as viperin is a member of the radical‐SAM superfamily of enzymes [10]. The RSAD2 gene is an interferon‐stimulated gene (ISG) whose expression is induced by type‐I, type‐II and type‐III interferons, directly by viruses and by LPS [11, 12, 13, 14, 15, 16, 17, 18]. It is known that expression of RSAD2 restricts replication of a wide range of RNA and DNA viruses in different cells [19, 20] and this effect is proposed to result from an altered metabolic state [21]. Biochemical and cell biological studies revealed that RSAD2 can catalyse the transformation of CTP to its 3'‐deoxy‐3',4'‐didehydro analogue (ddhCTP) [22]. Isotope labelling experiments [22], structural analysis [23] and biochemical studies [24] have also shed light on the enzymatic mechanism. Biochemical experiments showed that ddhCTP may act as a chain terminator of viral RNA‐dependent RNA polymerases (RdRps) (IC_50_> 20,000 µM) [22]. It should be noted that the reported IC_50_ values of ddhCTP as a chain terminator of viral RdRps were not corrected for the observed background effect of CTP on biochemical assays used to measure chain‐termination activity [25]. Nevertheless, if ddhCTP acts as a chain terminator of viral RdRps, the question that arises is why does then the cellular activity of RSAD2 affects many processes like glucose homeostasis [26] and expression of immune‐related genes [27]? To answer this fundamental question, metabolomic experiments using HEK293T cells and macrophages derived from human induced pluripotent stem cells (hiPSCs) were used. It was discovered that cellular activity of RSAD2 diminishes activity of NAD^+^‐dependent enzymes including that of GAPDH inside cells (Fig. 2) increasing intracellular levels of G3P and metabolites of pentose phosphate pathway [25, 28]. Subsequent biochemical studies confirmed that ddhCTP inhibits activity of GAPDH in a test tube with an IC50 value of 55.8 ± 0.2 µM [25]. This value is bout 400‐fold less than the reported IC_50_ value of ddhCTP as chain terminator and is less than the reported cellular concentration of ddhCTP (100–300 µM) [22]. These data suggest that under physiological conditions ddhCTP is more efficient in inhibiting GAPDH than acting as a chain terminator of RdRps.

### S‐nitrosylation

Nitric oxide (NO) has emerged as a key player in innate immune response to bacterial and viral pathogens [29, 30]. It is synthesized from L‐arginine by the catalytic function of the metalloenzyme nitric oxide synthase (NOS). In humans at least three isoforms of NOS have been reported (NOS‐I, NOS‐II and NOS‐III) [31, 32]. These metalloenzymes have binding sites for NADPH, FMN, FAD and calmodulin (CaM). The active site of all three isoforms has a haem cofactor and catalyses conversion of L‐arginine to NO and L‐citrulline in two steps [33]. Several reports have shown that NO induces S‐nitrosylation of GPADH, which inhibits its activity (Fig. 2) [34, 35, 36, 37, 38]. It is suggested that S‐nitrosylation of the active site thiol leads to nonenzymatic ADP‐ribosylation, which inactivates the protein [37].

## Inhibition of GAPDH and a broad‐spectrum antiviral response

**Fig. 3:**
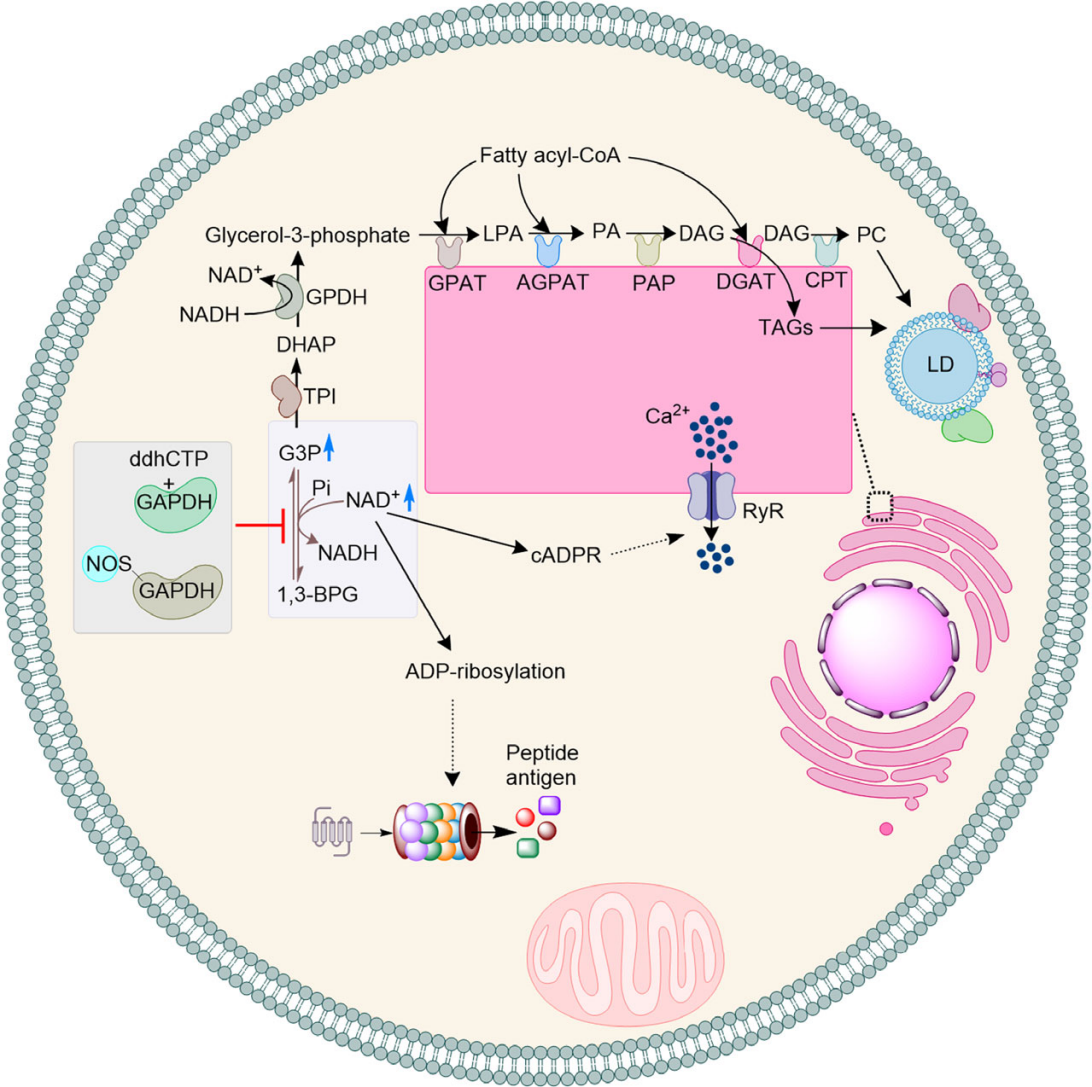
Inhibition of GAPDH increases the intracellular availability (blue arrow) of glyceraldehyde 3‐phosphate (G3P) and NAD^+^. An increase in the availability of G3P will support biosynthesis of TAGs and PC, which are the building blocks of lipid droplets (LDs). Increase in the availability of NAD^+^ will support synthesis of cADPR and ADP‐ribosylation. cADPR activates RyR receptor and induces release of Ca^2+^ from the cellular stores. ADP‐ribosylation can increase proteasomal activity and formation of peptide antigens.

Inhibition of GAPDH by ddhCTP or S‐nitrosylation will likely result in an increase in the cellular availability of NAD^+^. This will support protein ADP‐ribosylation and biosynthesis of cyclic ADP‐ribose (cADPR) [39, 40] (Fig. 3), both of which require NAD^+^ as a substrate. Consistently, it is shown that S‐nitrosylation of GAPDH and inhibition of its activity increases endogenous protein ADP‐ribosylation [34]. ADP‐ribosylation is shown to increase proteasomal activity [41, 42]. cADPR on the other hand, is a second messenger metabolite involved in modulation of Ca^2+^ signalling and homeostasis [43, 44, 45] (Fig. 3A). cADPR binds to ryanodine receptor (RyRs), which is expressed in many cell types including macrophages and T cells [46], and initiates the release of Ca^2+^ from the intracellular store (Fig. 3) [47, 48, 49]. Aligned with these observations, it has been shown that the cellular level of NAD^+^ controls Ca^2+^ store and release [50].

Inhibition of GAPDH can also increase the cellular availability of G3P. Consistently, macrophages expressing RSAD2, which can produce ddhCTP, show a higher intracellular level of G3P as compared to RSAD2‐KO macrophages [25]. Increase in the cellular availability of G3P supports biosynthesis of triacylglycerols (TAGs) and phosphatidylcholine (PC), which are the building blocks of lipid droplets (LDs) [51, 52]. G3P is converted to dihydroxyacetone phosphate (DHAP) by the catalytic activity of triosephosphate isomerase (Fig. 3). Subsequently, DHAP is converted by the NADH‐dependent activity of glycerol‐3‐phosphate dehydrogenase (GPDH) to glycerol‐3‐phosphate (Fig. 3). Next, in a series of enzymatic reactions [52], which have been studied since early 1950s and are localized at the cytosolic face of the endoplasmic reticulum (ER), glycerol‐3‐phosphate and fatty acyl‐CoA are combined to generate TAGs and PC. Consistently, using ^13^C‐labelling experiments it was found that upon formation of classically activated macrophages (M1 macrophages), in which expression of RSAD2 is highly induced [53], formation of LD increases and the carbon for the synthesis of LDs originates from G3P [54].

**Fig. 4:**
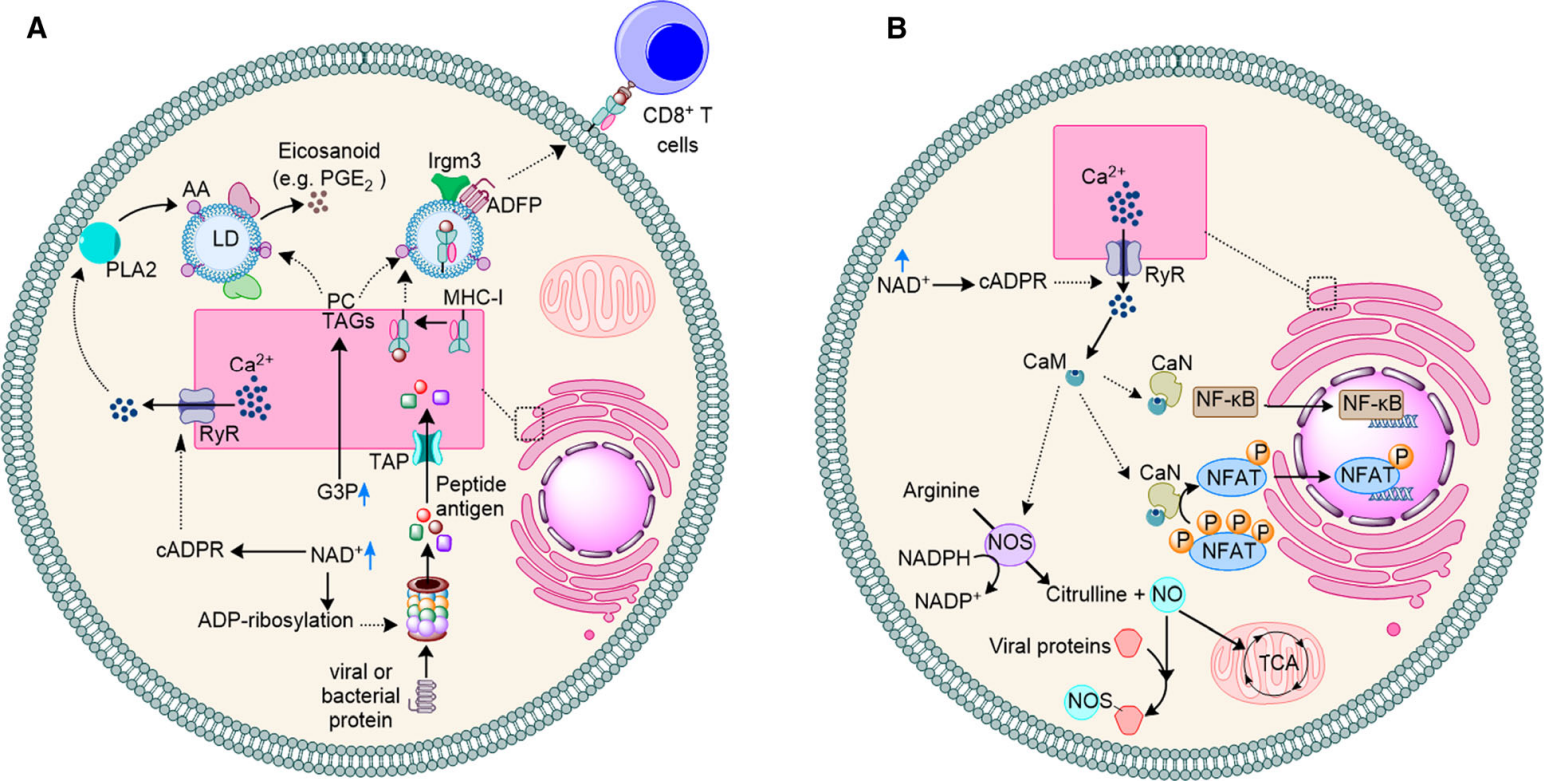
Inhibition of GAPDH and increase in the cellular availability of G3P and NAD^+^ support a broad‐spectrum immune response via at least four mechanisms. (a) Increase in the cellular availability of G3P and NAD^+^ supports eicosanoid synthesis and antigen cross‐presentation via MHC‐I. (b) Increase in the cellular availability of NAD^+^ will induce release of Ca^2+^ from the cellular stores and CaM‐dependent activation of NFAT and NF‐κB. On the other hand, CaM induces synthesis of NO by iNOS, which promotes S‐nitrosylation of viral proteins to restrict viral replication in immune cells like macrophages or modulates TCA cycle to induce formation of inflammatory macrophages.

Stimulation of proteasome activity by ADP‐ribosylation, cADPR‐dependent stimulation of Ca^2+^ release from the cellular stores, and an increase in biosynthesis of TAGs and PC, is likely to support a systemic immune response in cells in at least four ways (Fig. 4):

- Eicosanoids storm: Eicosanoids have a wide range of functions in inflammation and immune response to pathogens. Overall, available data suggest that eicosanoids can have both pro‐inflammatory and anti‐inflammatory activities depending on context and thus, may contribute to a balanced immune response [55]. It has been established that LDs are not just fat‐storing organelles and that they are important mediators of the innate immune response to pathogens [56]. LDs are shown to be a site for biosynthesis of eicosanoids [57]. Formation of eicosanoids occurs via a complex and highly regulated process [57] starting with liberation of arachidonic acid (AA) from phospholipids by Ca^2+^‐dependent phospholipases (PL)A_2_ [58, 59]. In different cells including innate immune cells like macrophages, the LDs are rich in AA [60, 61, 62, 63, 64]. Characterization of lipid droplets in different cells has revealed that enzymes involved in catalytic conversion of AA to eicosanoid like PGE_2_, namely cyclooxygenase 1 and 2 (COX1 and COX2), are localized to the LDs [65]. These data strongly suggest that LDs are at least partially involved in synthesis of inflammatory eicosanoids from AA and their downstream signalling pathways. Inhibition of GAPDH is likely to support LDs‐mediated eicosanoids biosynthesis in at least two ways: (i) increase in the cellular availability of G3P for biosynthesis of TAGs and PC and formation of LDs (Fig. 4A) and (ii) increase in the cellular availability of NAD^+^ and induction of cADPR‐dependent release of Ca^2+^ from cellular stores to support activity of the Ca^2+^‐dependent phospholipases PLA_2_ and liberation of AA (Fig. 4A).
- Antigen cross‐presentation via major histocompatibility class I: This process requires proteasomal activity and LDs. An increase in proteasomal activity increases the rate of formation of peptide antigens for cross‐presentation via major histocompatibility class I (MHC‐I) [66, 67, 68]. In many cell types including macrophages and DCs, a major path of antigen cross‐presentation involves transfer of peptide antigen into the endoplasmic reticulum (ER) lumen by the ATP‐dependent function of the TAP system (Fig. 4A) [69, 70]. In the ER lumen, peptide antigens bind to MHC‐1 and the complex is transported to the cell surface for presentation to CD8^+^ T cells. A mechanism of transportation to the cell surface is through LDs [56]. In dendritic cells (DCs), the immune‐related GTPs protein, namely Irgm3, localizes to the LDs [71]. When the gene expressing Irgm3 or adipose differentiation‐related protein (ADRP, also known as ADFP), which regulates LD biogenesis and dynamics [72, 73], was inactivated, formation of LDs was impaired and cross‐presentation of antigen to CD8^+^ T cells was abrogated [71]. Additionally, saponin‐based adjuvants (SBAs), which are used in cancer vaccines, induce formation of LDs in CD11b^+^ DCs and this increase causes a saponin‐dependent increase in antigen cross‐presentation and T‐cell activation [74]. Therefore, a concomitant increase in the cellular availability of G3P and NAD^+^ due to inhibition of GAPDH will ensure formation of LDs as carriers of the MHC‐I/antigen complex, and increase proteasomal activity via an ADP‐ribosylation pathway to provide the peptide antigens (Fig. 4A). Consistent with this mechanism, the cellular activity of RSAD2 (viperin), which generates the ddhCTP metabolite and inhibits GAPDH, stimulates degradation of Zika virus and tick‐borne encephalitis virus nonstructural protein NS3 via a proteasome‐dependent manner [75].
- NFAT‐ and NF‐κB‐mediated immune regulation. In innate immune cells like macrophages or T cells release of Ca^2+^ activates a range of immune defence mechanisms (Fig. 3B). Ca^2+^ binds to calmodulin (CaM) and the complex activates the phosphatase calcineurin (CaN) [76, 77]. In turn, CaN dephosphorylates and activates nuclear factor of activated T cells (NFAT) [78]. Additionally, CaN plays a role in LPS‐induced nuclear factor‐κB (NF‐κB) activation in macrophages [79, 80, 81]. NFAT‐ and NF‐κB regulate expression of several genes involved in immune cell response and function including IL‐10, IL‐6, IL‐8, IFN‐1, IFN‐2, TNF‐α and multiple TLR‐inducible genes including iNOS [82, 83, 84, 85, 86, 87, 88, 89, 90]. Thus, inhibition of GAPDH by ddhCTP or NO, and the likely increase in the cellular availability of NAD^+^, will modulate NFAT‐ or NF‐κB‐dependent expression of inflammatory genes. In summary, inhibition of GAPDH and an increase in the cellular level of NAD^+^ are likely to induce stimulation of cADPR and release of Ca^2+^. This will modulate activity of NFAT‐ and NF‐κB for a balanced and effective antiviral immune response. There is growing evidence in support of this mechanism. Firstly, when RSAD2 (viperin) gene was knocked out, thereby abrogating the inhibition of GAPDH by ddhCTP, the mRNA level of genes whose expression is regulated by NFAT or NF‐κB including iNOS and TNF‐α was affected in macrophages [91]. Secondly, NFAT and NF‐κB regulate Th2 response [92] and cellular activity of RSAD2 modulates activity of NF‐κB and AP1, which interact with NFAT, for optimal Th2 response [93]. Finally, overexpression of viperin upregulates expression of a wide range of immune‐related genes including IL‐8, IFN‐1 and IFN‐2 [27], whose expression is regulated by NFAT.
- Nitric oxide and immune response. In many cells, nitric oxide (NO) is an important product of the innate immune response and has broad‐spectrum antiviral and antibacterial activity [94, 95, 96, 97]. It can contribute to viral restriction via different mechanisms. NO causes S‐nitrosylation of different viral proteins, abolishes their activity and reduces viral replication [98] (Fig. 4B). Viral components, including proteases [99, 100, 101, 102], RNA‐dependent RNA polymerases (RdRps) [103], and transcriptase [104], are known to be inhibited by NO‐mediated S‐nitrosylation. Replication of a number of viruses is restricted by NO including herpes simplex virus type 1 [105], Japanese encephalitis virus [106], coxsackievirus [101], dengue virus type 2 (DNGV‐2) [103], influenza virus [107] and HIV‐1 [102, 104]. Additionally, NO can modulate mitochondrial metabolism to induce formation of inflammatory macrophages [108]. Therefore, inhibition of GAPDH and an increase in the cellular availability of NAD^+^, which will induce cADPR‐dependent Ca^2+^ release, will induce activity of iNOS via CaM binding (Fig. 4B). The resulting NO can support a broad‐spectrum antiviral response via S‐nitrosylation of viral protein or by further modulating the metabolism in immune cells like macrophages.

## Concluding remarks

In summary, we demonstrate that the glycolytic and housekeeping enzyme GAPDH is inhibited or modified by the metabolites, namely ddhCTP and NO, produced by two ISG protein products, RSAD2 and nitric oxide synthase, respectively. Inhibition of the NAD^+^‐dependent conversion of G3P by GAPDH supports several downstream metabolic and signalling pathways, specifically biosynthesis of TAGs and PC, which are precursors of LDs, protein ADP‐ribosylation and synthesis of cADPR. Together, these metabolites stimulate a balanced immune response via inflammatory eicosanoids, antigen cross‐presentation, activation of NFAT and NF‐κB and stimulation of formation of NO. This immunometabolic regulation of central carbon metabolism to stimulate a broad‐spectrum immune response provides an explanation for the wide range of effects observed due to expression of RSAD2 (viperin) in many cell types: these include the broad‐spectrum antiviral response [19], optimal Th2 cytokine production [93], which requires NFAT function [109], modulation of cellular lipid metabolism during human cytomegalovirus and influenza virus infections [110, 111], induction of type‐1 interferon production in plasmacytoid dendritic cells via a Toll‐like receptor‐mediated mechanism [112], interference with glucose homeostasis [26] and regulation of macrophage polarization [91].

Our analyses suggest that inhibition of GAPDH by the cellular innate immune response primes a broad‐spectrum immune response to viral infection. This is in opposed to recent reports [113, 114] suggesting that inhibition of GAPDH reduces immune response and thus, is a potential therapeutic approach for treating inflammatory diseases. These studies are based on use of small molecules such as the drug dimethyl fumarate (DMF) [113], which is used to treat autoimmune diseases, or 4‐octyl itaconate [114]. These molecules were suggested to directly modify Cys150 or Cys22, respectively, in GAPDH, and inhibit its activity. This inhibition was linked to a reduction in synthesis of inflammatory cytokines such as TNF‐α in T cells and macrophages with the assumption that no other protein in the cell was modified [113, 114]. In contrast to this assumption, analysis of global proteome in T cells reveals more than 2400 cysteine residues that could potentially be modified by DMF [115]. It was shown that two cysteine residues in protein kinase Cθ are target of modification by DMF and these modifications interfere with T‐cell activation [115].

Because the cellular innate immune response adopts mechanism that leads to inhibition of GAPDH, which as we discussed is likely to induce a broad‐spectrum immune response, we propose that in individuals with weakened cellular innate immune system inhibition of GAPDH might be a therapeutic approach to help prime the innate immune response via at least four mechanisms: (i) supporting formation of eicosanoids, (ii) assisting antigen cross‐presentation via MHC‐I, (iii) mediating immune response via NFAT and NF‐κB and (iv) stimulating synthesis of NO. Hence, we speculate that inhibition of GAPDH might help in the treatment of infection with viruses such as SARS‐CoV‐2. Future works should test the validity of this proposal.

## Conflict of interest

The authors declare no conflict of interest.

## Author contributions

KHE conceived the study and wrote the manuscript with contribution from all the authors.
